# Insecticide resistance: a challenge to malaria vector control in Ethiopia

**DOI:** 10.1186/1475-2875-11-S1-P139

**Published:** 2012-11-09

**Authors:** Meshesha Balkew, Alemayehu Getachew, Shelleme Chibsa, Dereje Olana, Richard Reithinger, William Brogdon

**Affiliations:** 1 Aklilu Lemma Institute of Pathobiology, Addis Ababa University, Addis Ababa, Ethiopia; 2 Research Triangle Institute International, Addis Ababa, Ethiopia; 3 U.S. Agency for International Development, Addis Ababa, Ethiopia; 4 World Health Organization, Addis Ababa, Ethiopia; 5 U.S. Center for Disease Control and Prevention, Atlanta, Georgia

## Background

In Ethiopia, indoor residual spraying (IRS) and insecticide-treated bed nets form the main malaria vector control. As the two tools rely on synthetic insecticides, it was found necessary to document the up-to-date distribution and levels of insecticide susceptibility of *Anopheles arabiensis*.

## Materials and methods

Between 2008 and 2011, insecticide susceptibility tests were carried out in 39 localities out of which 12 were repeatedly visited from 2 to 4 years. Tests were conducted using WHO test kits and procedures [1] on non-blood fed, 48-72 hours old female *An. arabiensis* which were reared from field collected larvae and pupae. The insecticides were discriminating doses of DDT, malathion, fenitrothion, primiphos-methyl, propoxur, bendiocarb, deltamethrin and lambdacyhalothrin. Controls were exposed to insecticide free oil impregnated papers. The WHO recommendations were applied to classify the population as susceptible, acquiring possible resistance and resistance [1]. The presence and frequency of the target site insensitive resistance mechanisms, *kdr* (L1014F mutation) and *ace-1* (G119S mutation) were investigated from vector populations of nine localities following the procedures described in [2,3].

## Results

All results depicted very low mortalities of *An. arabiensis* due to DDT, implicating wide distribution of resistance to this insecticide (Table 1). Resistance is also significantly high to deltamethrin, lambdacyhalothrin and malathion. Bendiocarb resistant populations were also detected from a few localities. The vector populations are susceptible to primiphos-methyl and propoxur, susceptibility was also very high to fenithrotion. Of 229 *An. arabiensis*, more than 95% were found to carry the *kdr* gene (both homozygous and heterozygous genotypes) while 47 tested specimens were without the *ace-1* allele mutation.

**Table 1:** Mortality results of *Anopheles arabiensis* and number of localities with susceptible and resistant populations (2008-2011)

Insecticide	Percentage mortality		**Number of localities with *An.arabiensis***		
	
	average	Range	Susceptible	Possible of resistance	Resistance
**DDT**	15.2	0-85.0	-	1	38
**Deltamethrin**	72.7	18.8-100	1	8	19
**Lambdacyhalotrhrin**	49.9	3.0-94.0	-	2	13
**Malathion**	86.4	38.0-100	7	15	8
**Fenithrotion**	98.2	76.5-100	17	3	-
**Primiphos-methyl**	100	100	4	-	-
**Propoxur**	99.5	96.0-100	12	-	-
**Bendiocarb**	95.0	53.0-100	17	8	2

## Conclusions

Similar studies in the past by other workers [4-7] together with this one showed increased resistance of *An. arabiensis* to insecticides belonging to the four major classes. This would pose a serious challenge to vector control in the coming years. Given the small number of insecticides for IRS and LLINs, the Federal Ministry of Health of Ethiopia should take timely measure by formulating a policy as well as implementing insecticide resistance management within the frame work of integrated vector management.
